# Psychometric Evaluation of the Turkish Version of the Dental Fear Survey

**DOI:** 10.1016/j.identj.2026.109774

**Published:** 2026-08-01

**Authors:** Fatih Oluş, Hüseyin Babun

**Affiliations:** Department of Oral and Maxillofacial Surgery, Faculty of Dentistry, Akdeniz University, Antalya, Türkiye

**Keywords:** Dental fear, Dental Fear Survey, Dental anxiety, Psychometric validation, Turkish version, Reliability and validity

## Abstract

**Introduction and aims:**

Dental fear is an important barrier to dental attendance and may affect oral health behaviours, treatment acceptance and patient-centred care. The Dental Fear Survey (DFS) is a widely used multidimensional instrument. Although the DFS has previously been used in Turkish populations, comprehensive psychometric evidence for a systematically adapted Turkish version in adult clinical patients has remained limited. This study aimed to translate and culturally adapt the DFS into Turkish and evaluate its psychometric properties.

**Methods:**

The DFS was translated and culturally adapted following established guidelines. The prefinal version was pilot-tested in 30 adult dental patients, and content validity was evaluated by 10 experts. The main evaluation included 502 adult dental patients, divided into independent EFA (n = 253) and CFA (n = 249) subsamples. Internal consistency, 2-week test–retest reliability in 50 patients and convergent validity with the Turkish Modified Dental Anxiety Scale were assessed.

**Results:**

EFA suggested a meaningful 3-factor structure consisting of dental avoidance and anticipatory anxiety, physiological arousal and fear of specific dental stimuli and procedures, explaining 66.99% of the variance. CFA showed improved but limited fit after theoretically justified modifications; therefore, the 3-factor structure was interpreted cautiously. Internal consistency was excellent (Cronbach’s alpha = 0.95 for the total scale; 0.81-0.95 for subscales). Test–retest reliability was also excellent (ICC = 0.98 for the total score; 0.96-0.98 for subscales). The total DFS score correlated strongly with the MDAS score (r = 0.83, *P* < .001).

**Conclusion:**

The Turkish DFS demonstrated favourable psychometric properties, particularly for internal consistency, temporal stability and convergent validity. Its multidimensional structure was meaningful, although structural validity should be interpreted cautiously and confirmed in independent samples.

**Clinical relevance:**

The Turkish DFS may facilitate clinicians identify components of dental fear and support patient communication and assessment in Turkish-speaking adults.

## Background

Dental fear is a prevalent and clinically significant condition that negatively influences oral health behaviours, patterns of dental attendance and treatment outcomes.^1^, ^2^, ^3^ Individuals experiencing high levels of dental fear are more likely to delay or avoid dental visits, often seeking care only when pain or functional impairment becomes unavoidable, which frequently results in the need for more invasive and complex dental procedures.^2^^,^^4^ This avoidance–escalation cycle has been shown to contribute to poorer oral health–related quality of life and increased treatment burden for both patients and clinicians.^3^^,^^5^ Moreover, dental fear presents a challenge for dental professionals by complicating patient cooperation, prolonging treatment time and potentially compromising clinical outcomes.^6^ Consequently, the accurate identification and assessment of dental fear is widely recognised as an essential component of contemporary dental practice and oral health research.^1^^,^^7^ More broadly, recent oral health research has also highlighted the value of structured clinical and psychosocial parameters for improving prediction, comparability and clinical interpretation in dental research.^8^

Several self-report instruments have been developed to assess dental fear and anxiety in clinical and research settings. Widely used measures such as the Dental Anxiety Scale (DAS) and its modified version, the Modified Dental Anxiety Scale (MDAS), primarily focus on anticipatory anxiety related to dental visits and specific procedures, providing a brief and practical screening of overall dental anxiety levels.^9^^,^^10^ While these instruments are valuable for rapid assessment, they offer limited insight into the multidimensional nature of dental fear. In contrast, the Dental Fear Survey (DFS), originally developed by Kleinknecht and colleagues, was designed to capture multiple dimensions of dental fear, including avoidance behaviours, physiological arousal and fear elicited by specific dental stimuli and situations.^11^ Owing to its comprehensive structure, the DFS has been widely used in both clinical and epidemiological studies and has demonstrated robust psychometric properties across diverse populations.^12^, ^13^, ^14^ However, cultural and linguistic differences may influence how dental fear is perceived and reported, underscoring the need for language-specific validation studies to ensure the reliability and validity of the instrument in different cultural contexts.^15^

Although the DFS has previously been used in Turkish populations and earlier studies have provided initial psychometric observations, a comprehensive Turkish validation study based on a systematic cross-cultural adaptation process and including content validity, pilot testing, exploratory and confirmatory factor analyses, internal consistency, test–retest reliability and convergent validity in an adult clinical dental population has remained limited. Therefore, although dental anxiety has been investigated in Türkiye using various instruments, the limited availability of a comprehensively validated multidimensional Turkish DFS has restricted both clinical assessment and research comparability. Moreover, advances in psychometric methodology emphasise the importance of systematic cross-cultural adaptation procedures, including pilot testing and evaluation of measurement properties, to ensure the validity and interpretability of self-report instruments across different languages and cultural settings.^15^^,^^16^ Recent oral health questionnaire validation studies have similarly emphasised the need for culturally adapted and psychometrically tested patient-reported instruments in specific clinical populations.^17^ Establishing a validated Turkish version of the DFS may also contribute to cross-cultural comparability in dental fear research and support standardised assessment in Turkish-speaking clinical and community populations. Therefore, the aim of the present study was to translate and culturally adapt the DFS into Turkish and to evaluate its psychometric properties in a clinical adult population. We hypothesised that the Turkish version of the DFS would retain a theoretically meaningful 3-factor structure consistent with the multidimensional construct of dental fear, demonstrate high internal consistency and temporal stability and show moderate-to-strong positive correlations with the Turkish version of the MDAS, supporting convergent validity. Construct validity was examined by assessing the relationship between the Turkish version of the DFS and the Turkish version of the MDAS, which has previously demonstrated acceptable validity and reliability in the Turkish population.^18^

## Materials and methods

### Study design and participants

This methodological study was designed to translate, culturally adapt and psychometrically evaluate the DFS for use in the Turkish adult population. The translation and cross-cultural adaptation process followed the guideline proposed by Beaton et al.,^15^ and the psychometric evaluation was structured using measurement property terminology consistent with COSMIN recommendations.^16^ Data were collected between January and April 2026 from adult patients who presented for dental care at the Faculty of Dentistry of Akdeniz University. Eligible patients who attended the clinic during the study period were approached consecutively and invited to participate. Therefore, the study used a consecutive convenience sampling approach based on voluntary participation. Individuals aged 18 years and older who were able to read and understand Turkish were eligible to participate. Patients with cognitive impairment or psychiatric conditions that could interfere with questionnaire completion were excluded.

Sample size adequacy was evaluated according to commonly used recommendations for scale validity and reliability studies.^19^ For factor analytic procedures, a sample size corresponding to at least 5 to 10 participants per item is generally considered acceptable. Because the DFS consists of 20 items, the minimum recommended sample size would range from 100 to 200 participants. The main validation sample in the present study included 502 participants, corresponding to approximately 25 participants per item. In addition, the sample was divided into 2 independent subsamples for EFA (n = 253) and CFA (n = 249), each exceeding the recommended minimum participant-to-item ratio. Therefore, the sample size was considered adequate for the planned psychometric analyses.

The main validation sample was then divided into 2 independent subsamples. Exploratory factor analysis (EFA) was performed using data from 253 participants, whereas confirmatory factor analysis (CFA) was conducted in an independent subsample of 249 participants. The total sample included in the main psychometric analyses was therefore 502 participants. In addition, the prefinal Turkish version was pilot-tested in a separate group of 30 adult dental patients, and test–retest reliability was assessed in another separate subgroup of 50 adult dental patients. Participants included in the pilot testing and test–retest reliability assessment were not included in the EFA or CFA samples.

Participation was voluntary, and all participants provided written informed consent prior to enrolment.

### Instrument (Dental Fear Survey)

The DFS is a self-report questionnaire originally developed by Kleinknecht and colleagues to assess dental fear as a multidimensional construct. The instrument consists of 20 items designed to evaluate different aspects of dental fear, including avoidance behaviours, physiological arousal and fear elicited by specific dental stimuli and situations.

In accordance with the original scale structure, the DFS items are organised into two response formats. Items 1 to 7 assess the frequency of fear-related reactions and behaviours associated with dental treatment, using a 5-point Likert scale ranging from never (one point) to nearly every time (five points). Items 8 to 20 evaluate the intensity of fear and anxiety elicited by specific dental situations and procedures, using a 5-point Likert scale ranging from very relaxed (one point) to so anxious that one feels ill (five points). Higher total scores indicate greater levels of dental fear.

The total DFS score is calculated by summing all item responses, with possible scores ranging from 20 to 100. The DFS has been widely used in clinical and epidemiological research and has demonstrated strong psychometric properties across diverse cultural contexts.

The Turkish DFS was administered using the original 20-item order and scoring structure of the English version. The total score was calculated by summing all item responses, with higher scores indicating greater dental fear. Subscale scoring and interpretation for the Turkish version were subsequently determined based on the factor structure identified through exploratory factor analysis.

### Translation and cross-cultural adaptation

The translation and cross-cultural adaptation of the DFS into Turkish were conducted in accordance with the guideline proposed by Beaton et al.^15^ The process included forward translation, synthesis of the translations, back translation, expert committee review, pilot testing of the prefinal version and final approval of the Turkish version.

Initially, the original English version of the DFS was independently translated into Turkish by 2 bilingual translators with expertise in dentistry and health sciences. The two forward translations were compared and synthesised into a single preliminary Turkish version through consensus, with particular attention to semantic, idiomatic, experiential and conceptual equivalence.

The synthesised Turkish version was then back translated into English by bilingual translators who were blinded to the original version of the instrument. The back-translated version was compared with the original DFS to identify possible discrepancies in meaning. Any differences were discussed by the research team and resolved through consensus.

The prefinal Turkish version was reviewed by an expert committee consisting of professionals experienced in dentistry, psychometrics and clinical research. The committee evaluated the clarity, relevance, cultural appropriateness and conceptual equivalence of each item. Minor linguistic refinements were made where necessary to improve comprehensibility while preserving the original meaning and structure of the scale. No items were added, removed, or conceptually modified during the adaptation process.

### Pilot testing

The prefinal Turkish version of the DFS was pilot-tested in a separate group of 30 adult dental patients who were not included in the main psychometric analyses. The aim of the pilot testing was to evaluate comprehensibility, clarity and cultural relevance. Participants were asked whether any item was difficult to understand, ambiguous, or culturally inappropriate. Feedback obtained during pilot testing was reviewed by the research team. Minor wording refinements were made when necessary to improve clarity; however, no item was removed or conceptually modified. The pilot testing confirmed that the Turkish DFS was understandable and suitable for use in the target adult dental patient population.

### Content validity (CVI)

Content validity was evaluated by a panel of 10 professionals with complementary expertise: 2 oral and maxillofacial surgery specialists, 1 restorative dentistry/endodontics specialist, 2 periodontology specialists, 1 psychiatrist or clinical psychologist, 1 public health specialist, 1 biostatistician and 2 anaesthesiologists experienced in dental sedation and anxiety management. All experts had professional or academic experience relevant to dental fear, dental treatment-related anxiety, clinical research, or psychometric evaluation.

Each expert independently reviewed the prefinal Turkish version of the DFS and evaluated each item in terms of relevance, clarity, cultural appropriateness and conceptual equivalence using a 4-point Likert scale. For content validity analysis, item relevance ratings were used to calculate the item-level content validity index (I-CVI), and the scale-level content validity index based on the average method (S-CVI/Ave) was calculated. An I-CVI value of 1.00 indicated that all experts rated the item as relevant. All items achieved an I-CVI of 1.00, and the S-CVI/Ave was 1.00, indicating excellent content validity. Based on expert feedback, a minor linguistic revision was made to 1 item to improve cultural clarity while preserving the original structure and conceptual meaning of the scale.

### Data collection procedure

Data collection was carried out between January and April 2026 in the high-volume clinical setting of the Faculty of Dentistry of Akdeniz University. Within this 4-month study window, the participant-related phases of the study were completed in a sequential and partially overlapping manner. The prefinal Turkish version was first pilot-tested, followed by recruitment for the main validation sample. During the main data collection period, eligible patients were approached consecutively, which allowed recruitment of the EFA and CFA subsamples within the same study window. Participants included in the test–retest analysis completed the questionnaire on 2 occasions separated by a 2-week interval. The preceding translation, back-translation and expert committee procedures were completed before participant recruitment.

Eligible participants were approached during their routine dental visit and were informed about the purpose and voluntary nature of the study. After providing written informed consent, participants completed the questionnaires prior to dental treatment in a quiet area of the clinic to minimise external distractions.

All questionnaires were administered in paper-and-pencil format and completed under the supervision of a trained researcher, without any time restrictions. Participants were instructed to respond to all items independently, and no guidance or interpretation was provided during questionnaire completion, except for clarification of procedural questions when necessary. For participants with reading difficulties, items were read aloud verbatim by the researcher without additional explanation or interpretation.

The data collection set included a demographic information form, a dental history and trigger questionnaire, the Turkish version of the DFS and the previously validated Turkish version of the MDAS. Completed questionnaires were checked for completeness immediately after submission, and no identifying personal information was recorded to ensure participant confidentiality.

### Psychometric evaluation

The psychometric properties of the Turkish version of the DFS were evaluated in accordance with established methodological recommendations for patient-reported outcome measures. Analyses were conducted separately for exploratory and confirmatory purposes using 2 independent subsamples.

#### Structural validity

Exploratory factor analysis (EFA) was performed on the first subsample (n = 253) to examine the underlying factor structure of the Turkish DFS. The suitability of the data for factor analysis was assessed using the Kaiser–Meyer–Olkin (KMO) measure of sampling adequacy and Bartlett’s test of sphericity. Factor extraction was conducted using principal component analysis, followed by Varimax rotation. The number of factors was determined by eigenvalues greater than 1.0, scree plot inspection and theoretical interpretability.

Confirmatory factor analysis (CFA) was conducted on the second independent subsample (n = 249) to test the factor structure identified in the EFA. Model fit was evaluated using multiple goodness-of-fit indices, including the chi-square statistic (χ²), the comparative fit index (CFI), the Tucker–Lewis index (TLI), the root mean square error of approximation (RMSEA) and the standardised root mean square residual (SRMR). Acceptable model fit was defined according to commonly accepted criteria.

#### Internal consistency

Internal consistency of the DFS total scale and its subscales was assessed using Cronbach’s alpha coefficients. Values of α ≥ 0.70 were considered indicative of acceptable internal consistency.

#### Test–retest reliability

Test–retest reliability was assessed in a separate subgroup of 50 adult dental patients who were not included in the pilot, EFA, or CFA samples. Eligible patients were invited consecutively to participate in the test–retest phase if they agreed to complete the DFS again after 2 weeks and could be reached for the second assessment. The 2-week interval was selected to reduce recall bias while minimising the likelihood of true change in dental fear. At the second assessment, participants were asked whether they had undergone any dental treatment or experienced any frightening or painful dental event during the interval, as such experiences could potentially alter fear levels.

#### Construct validity

Construct validity was evaluated by examining convergent validity through correlations between the Turkish DFS scores and scores obtained from the Turkish version of the MDAS. Correlation analyses were performed using Pearson’s correlation coefficient. A priori, we hypothesised that DFS scores would show moderate-to-strong positive correlations with MDAS scores, supporting convergent validity.

### Statistical analysis

Statistical analyses were performed using SPSS v25 and AMOS v23 (IBM). Descriptive statistics were calculated to summarise participant characteristics and item-level responses, including means, standard deviations, frequencies and percentages, as appropriate. The normality of continuous variables was assessed using visual inspection and distributional indices.

Before conducting the factor analytic procedures, outliers were examined using boxplot analysis of the total DFS scores. Observations identified as extreme values outside the expected distribution of the total DFS score were reviewed, and 12 cases were excluded from the dataset before the EFA and CFA procedures. This step was performed to minimise the potential influence of extreme total-score response patterns on the exploratory factor solution and confirmatory model fit estimates.

EFA and CFA were conducted as described above using separate subsamples. Internal consistency was evaluated using Cronbach’s alpha coefficients. Test–retest reliability was assessed using intraclass correlation coefficients (ICC). Construct validity was examined through Pearson correlation analysis between the total and subscale scores of the Turkish DFS and the Turkish version of the MDAS.

All statistical tests were 2-tailed, and a *P*-value <.05 was considered statistically significant. Missing data were minimal and handled using complete-case analysis.

### Ethical considerations

The study was conducted in accordance with the ethical principles of the Declaration of Helsinki. Ethical approval was obtained from the Akdeniz University Clinical Research Ethics Committee (TBAEK-1205) prior to the initiation of the study. All participants were informed about the purpose, procedures and voluntary nature of the research, and written informed consent was obtained from each participant before data collection. Participation was entirely voluntary, and participants were informed of their right to refuse participation or withdraw from the study at any time without any consequences for their dental care.

The study was noninterventional and based solely on self-report questionnaires. No personal identifying information was collected, and all data were anonymised to ensure confidentiality. The collected data were used exclusively for scientific purposes.

## Results

### Participant characteristics

A total of 502 adult dental patients were included in the main psychometric analyses after the exclusion of 12 outliers identified by boxplot analysis of the total DFS scores before the factor analytic procedures. The main validation sample was divided into 2 independent subsamples: 253 participants for exploratory factor analysis (EFA) and 249 participants for confirmatory factor analysis (CFA).

In the EFA subsample, 138 participants were female (54.5%) and 115 were male (45.5%). The mean age was 35.70 ± 15.48 years, with a median age of 29 years and an age range of 18 to 83 years. In the CFA subsample, 135 participants were female (54.2%) and 114 were male (45.8%). The mean age was 31.62 ± 12.89 years, with a median age of 26 years and an age range of 18 to 73 years.

In the EFA subsample, 66.0% of participants reported that their last dental visit had occurred within the previous 6 months, whereas this proportion was 58.6% in the CFA subsample. A previous frightening or painful dental treatment experience was reported by 45.5% of participants in the EFA subsample and 52.2% in the CFA subsample. General fear of dental treatment, at least to some degree, was reported by 67.6% of participants in the EFA subsample and 74.7% in the CFA subsample.

Demographic and dental history characteristics of the EFA and CFA subsamples are presented in Table 1.

**Table 1:** Demographic and dental history characteristics of the EFA and CFA subsamples.

		EFA subsample		CFA subsample	
		n	%	n	%
**Sex**	Female	138	54.5	135	54.2
	Male	115	45.5	114	45.8
Educational status	Literate	2	0.8	12	4.8
	Primary education	23	9.1	28	11.2
	High school	66	26.1	53	21.3
	University	141	55.7	117	47.0
	Postgraduate	21	8.3	39	15.7
Previous frightening or painful dental treatment experience	No	138	54.5	119	47.8
	Yes	115	45.5	130	52.2
General fear of dental treatment	None	82	32.4	63	25.3
	Mild	111	43.9	114	45.8
	Moderate	42	16.6	55	22.1
	Severe	18	7.1	17	6.8

### Pilot testing and content validity

The prefinal Turkish version of the DFS was pilot-tested in a separate group of 30 adult dental patients. No major difficulty in comprehension or cultural interpretation was reported during pilot testing. Minor wording refinements were made to improve clarity, but no item was removed or conceptually modified.

Content validity was evaluated by a panel of 10 professionals with complementary clinical and methodological expertise, including dentistry, dental sedation and anxiety management, psychiatry or clinical psychology, public health and biostatistics. All experts independently rated each item as relevant, resulting in an item-level content validity index of 1.00 for all items and a scale-level content validity index based on the average method of 1.00, indicating excellent content validity.

### Exploratory factor analysis

The Turkish DFS was administered using the original 20-item order and total scoring structure of the English version. Subscale scoring and interpretation for the Turkish version were subsequently determined based on the factor structure identified through EFA. The final Turkish version of the DFS used in this study is provided as Supplementary File 1.

Before conducting EFA, the suitability of the data for factor analysis was evaluated. The Kaiser–Meyer–Olkin value was 0.92, indicating excellent sampling adequacy. Bartlett’s test of sphericity was statistically significant (χ² = 4218.00, *P* < .001), confirming that the correlation matrix was suitable for factor analysis.

Communality values ranged from 0.40 to 0.81, indicating that all items contributed adequately to the factor solution. The lowest communality value was observed for item 6, and all values were above the commonly accepted threshold of 0.30.

**Fig. 1:**
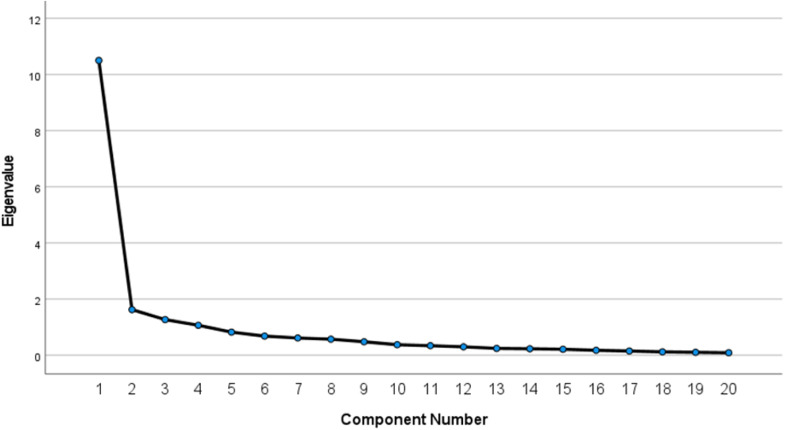
Scree plot of the exploratory factor analysis for the Turkish DFS.

The scree plot also supported examination of a 2- or 3-factor solution, with the 3-factor model retained based on theoretical interpretability and item distribution (Figure 1).

Principal component analysis with varimax rotation supported a 3-factor structure. The factor loadings and explained variance values are presented in Table 2. The 3 factors together explained 66.99% of the total variance. Factor loadings ranged from 0.55 to 0.85, indicating adequate to strong item loadings. No item showed inadequate communality or problematic cross-loading requiring item removal.

**Table 2:** EFA results of the Turkish DFS: factor loadings and explained variance.

Factor/Item	Factor loadings*	Explained variance
Factor 1: Dental avoidance and anticipatory anxiety 1. Has fear of dental work ever caused you to put off making an appointment?	0.62	16.55
2. Has fear of dental work ever caused you to cancel or not appear for an appointment?	0.59	
8. Making an appointment for dentistry	0.74	
9. Approaching the dentist’s office	0.79	
10. Sitting in the waiting room	0.62	

Factor 2: Physiological arousal during dental treatment 3. My muscles become tense	0.71	18.09
4. My breathing rate increases	0.78	
5. I perspire	0.77	
6. I feel nauseated and sick to my stomach	0.55	
7. My heart beats faster	0.69	

Factor 3: Fear of specific dental stimuli and procedures 11. Being seated in the dental chair	0.67	32.35
12. The smell of the dentist’s office	0.72	
13. Seeing the dentist walk in	0.67	
14. Seeing the anaesthetic needle	0.75	
15. Feeling the needle injected	0.80	
16. Seeing the drill	0.82	
17. Hearing the drill	0.85	
18. Feeling the vibrations of the drill	0.80	
19. Having your teeth cleaned	0.60	
20. All things considered, how fearful are you of having dental work done?	0.63	

Kaiser-Meyer-Olkin = 0.92	Total explained variance 66.99	
Bartlett’s test of sphericity; X^2^ = 4218.00 *P* < .001*		

⁎ Varimax rotation.

The first factor consisted of items 1, 2, 8, 9 and 10 and was interpreted as dental avoidance and anticipatory anxiety. This factor explained 16.55% of the total variance. The second factor consisted of items 3, 4, 5, 6 and 7 and was interpreted as physiological arousal during dental treatment. This factor explained 18.09% of the total variance. The third factor consisted of items 11 to 20 and was interpreted as fear of specific dental stimuli and procedures. This factor explained the largest proportion of variance, accounting for 32.35% of the total variance.

### Descriptive statistics of the Turkish DFS and subscales

Descriptive statistics for the Turkish DFS and its EFA-derived subscales are presented in Table 3. The mean total DFS score was 34.09 ± 14.14, with a median score of 29 and an observed range of 20 to 76. The mean score was 7.34 ± 3.08 for dental avoidance and anticipatory anxiety, 8.28 ± 3.94 for physiological arousal during dental treatment and 18.46 ± 8.74 for fear of specific dental stimuli and procedures.

**Table 3:** Descriptive statistics of the Turkish DFS total score and EFA-derived subscales.

Scale/Subscale	Min	Max	Mean ± SD	Median
Dental avoidance and anticipatory anxiety	5.00	21.00	7.34 ± 3.08	6.00
Physiological arousal during dental treatment	5.00	21.00	8.28 ± 3.94	7.00
Fear of specific dental stimuli and procedures	10.00	50.00	18.46 ± 8.74	16.00
Total DFS score	20.00	76.00	34.09 ± 14.14	29.00

DFS, Dental Fear Survey; EFA, exploratory factor analysis; SD, standard deviation.

### Internal consistency and item-total correlations

The Turkish DFS demonstrated excellent internal consistency. Cronbach’s alpha was 0.95 for the total scale. Subscale reliability was also high, with Cronbach’s alpha values of 0.81 for dental avoidance and anticipatory anxiety, 0.87 for physiological arousal during dental treatment, and 0.95 for fear of specific dental stimuli and procedures. Internal consistency coefficients for the Turkish DFS total scale and EFA-derived subscales are presented in Table 4.

**Table 4:** Internal consistency reliability of the Turkish DFS total scale and EFA-derived subscales.

Scale/Subscale	Number of items	Cronbach’s alpha
Dental avoidance and anticipatory anxiety	5	0.81
Physiological arousal during dental treatment	5	0.87
Fear of specific dental stimuli and procedures	10	0.95
Total DFS score	20	0.95

DFS, Dental Fear Survey; EFA, exploratory factor analysis.

Corrected item-total correlations ranged from 0.32 to 0.84. The lowest corrected item-total correlation was observed for item 2, but this value remained within an acceptable range. Deletion of any item did not result in a meaningful improvement in the overall Cronbach’s alpha, supporting retention of all 20 items.

Split-half reliability analysis also supported internal consistency. Cronbach’s alpha values were 0.89 for the first half of the scale and 0.95 for the second half. The correlation between the 2 halves was 0.76, with a Spearman–Brown coefficient of 0.86 and a Guttman split-half coefficient of 0.84.

### Test–retest reliability

Test–retest reliability was evaluated in a separate subgroup of 50 adult dental patients who completed the Turkish DFS twice at a 2-week interval. The ICC for the total DFS score was 0.98 (95% CI: 0.97-0.99, *P* < .001), indicating excellent temporal stability. ICC values for the EFA-derived subscales were also excellent: 0.96 (95% CI: 0.93-0.98) for dental avoidance and anticipatory anxiety, 0.98 (95% CI: 0.97-0.99) for physiological arousal during dental treatment and 0.98 (95% CI: 0.96-0.99) for fear of specific dental stimuli and procedures (all *P* < .001).

### Convergent validity

Convergent validity was evaluated by examining correlations between the Turkish DFS and the Turkish version of the MDAS. The total DFS score showed a strong positive correlation with the MDAS score (r = 0.83, *P* < .001). Significant positive correlations were also observed between the MDAS and all EFA-derived DFS subscales. The correlation coefficients were 0.67 for dental avoidance and anticipatory anxiety, 0.64 for physiological arousal during dental treatment, and 0.80 for fear of specific dental stimuli and procedures, all with *P* < .001. Correlations between the Turkish DFS scores and MDAS scores are presented in Table 5. These findings supported the convergent validity of the Turkish DFS.

**Table 5:** Convergent validity of the Turkish DFS: correlations with the MDAS.

Scale/Subscale	MDAS	
	r	*P*
Dental avoidance and anticipatory anxiety	0.67	<.001*
Physiological arousal during dental treatment	0.64	<.001*
Fear of specific dental stimuli and procedures	0.80	<.001*
Total DFS score	0.83	<.001*

DFS, Dental Fear Survey; MDAS, Modified Dental Anxiety Scale.

⁎ *P* < .05

### Confirmatory factor analysis

CFA was conducted in an independent subsample of 249 participants to test the 3-factor structure identified in the EFA. The initial model showed suboptimal fit indices: CMIN = 915.81, CMIN/df = 5.48, RMSEA = 0.13, NFI = 0.78, CFI = 0.82, IFI = 0.82 and TLI = 0.80. Fit indices for the initial and modified CFA models are presented in Table 6.

**Table 6:** CFA fit indices of the Turkish DFS 3-factor model.

Initial model	CMIN(X^2^)	CMIN/df	RMSEA	NFI	CFI	IFI	TLI	
	915.81	5.48	0.13	0.78	0.82	0.82	0.80	

Modified model	CMIN(X^2^)	CMIN/df	RMSEA	NFI	CFI	IFI	TLI	SRMR

	607.60	3.73	0.10	0.86	0.90	0.90	0.90	0.07

CFA, confirmatory factor analysis; CFI, comparative fit index; CMIN, minimum discrepancy; IFI, incremental fit index; NFI, normed fit index; RMSEA, root mean square error of approximation; SRMR, standardised root mean square residual; TLI, Tucker–Lewis index.

Model fit was improved by adding error covariances suggested by modification indices only when the item pairs were also considered theoretically justifiable. Error covariance was specified between items 1 and 2 because both items assess avoidance-related behavioural consequences of dental fear, namely delaying an appointment and cancelling or not attending an appointment. Error covariance between items 12 and 13 was considered theoretically plausible because both items reflect anticipatory sensory and contextual cues within the dental clinic environment, including smelling the dental office and seeing the dentist enter the room. Similarly, items 14 and 15 both refer to the same invasive stimulus, the anaesthetic needle, representing visual exposure to the needle and the physical sensation of needle insertion. Finally, items 16 and 17 both involve the dental drill, capturing visual and auditory aspects of the same procedure-related stimulus. These modifications were therefore retained because they were supported by both statistical modification indices and conceptual overlap between item contents.

After these modifications, model fit improved compared with the initial model; however, the overall fit remained limited. The modified model yielded the following indices: CMIN = 607.60, CMIN/df = 3.73, RMSEA = 0.10, NFI = 0.86, CFI = 0.90, IFI = 0.90, TLI = 0.90 and SRMR = 0.07. In particular, the RMSEA value of 0.10, NFI value of 0.86 and CMIN/df value of 3.73 indicate that the CFA findings should be interpreted cautiously.

**Fig. 2:**
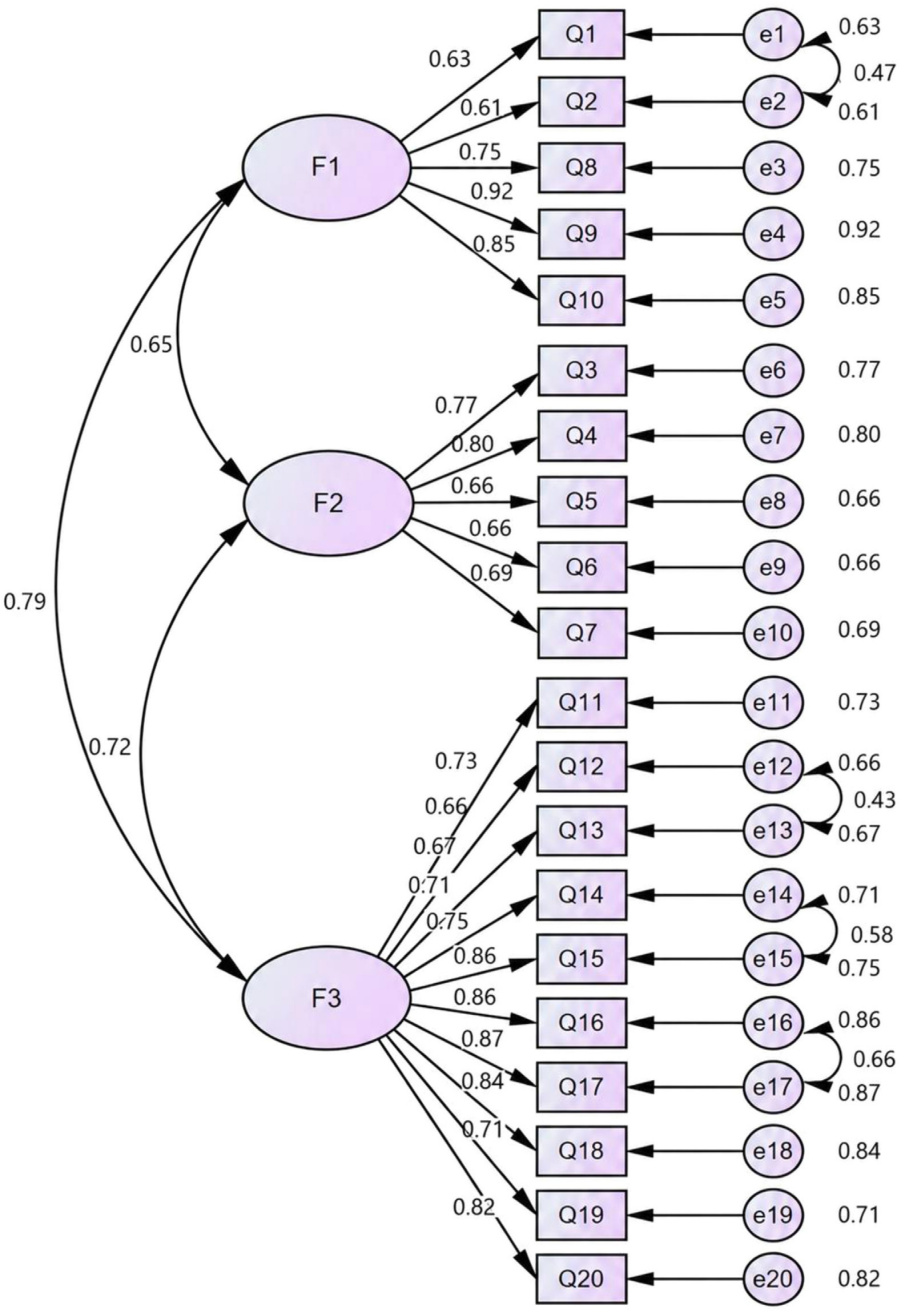
Final modified CFA model of the Turkish DFS 3-factor structure.

Overall, the CFA findings provided cautious and partial support for the EFA-derived 3-factor structure of the Turkish DFS, and further confirmation in independent samples is warranted. The final modified CFA model is shown in Figure 2.

## Discussion

The present study translated, culturally adapted and psychometrically evaluated the Turkish version of the DFS in an adult clinical dental population. The main findings indicate that the Turkish DFS demonstrated favourable psychometric properties, particularly in terms of content validity, internal consistency, test–retest reliability and convergent validity. The exploratory factor analysis suggested a theoretically meaningful 3-factor structure; however, the confirmatory factor analysis provided only cautious and partial support for this structure because the initial model showed poor fit and the RMSEA value remained at 0.10 after modification. Therefore, the structural validity findings should be interpreted with caution and require further confirmation in independent Turkish samples.

The factor analytic findings suggested a 3-factor structure for the Turkish DFS. Although the EFA indicated a theoretically interpretable solution, the CFA findings showed that model fit improved after theoretically justified modifications but remained limited, particularly with respect to the RMSEA value. The first factor, consisting of items 1, 2, 8, 9 and 10, reflected dental avoidance and anticipatory anxiety. The second factor, consisting of items 3–7, reflected physiological arousal during dental treatment. The third factor, consisting of items 11–20, reflected fear of specific dental stimuli and procedures. This structure is broadly consistent with the original factor analytic work by Kleinknecht et al., who identified 3 stable and reliable dimensions of the DFS: dental avoidance and anticipatory anxiety, fear associated with specific dental stimuli and procedures, and felt physiological arousal during dental treatment.^11^ In that original work, the DFS was proposed as a broader multi-item measure capable of sampling dental avoidance, somatovisceral arousal and fear responses to clinical dental situations more comprehensively than single-item fear ratings or shorter dental anxiety scales.^11^

The 3-factor structure observed in the present study is also consistent with several cross-cultural validation studies. The Japanese validation study reported that the DFS was designed to assess avoidance, physiological arousal and fear of specific dental situations and structural modelling confirmed the 3-factor structure underlying the English version.^20^ Similarly, the Lebanese Arabic validation study identified a 3-factor structure reflecting fear associated with specific dental stimuli and procedures, patterns of dental avoidance and anticipatory anxiety and physiological arousal during dental treatment.^21^ More recently, the Vietnamese version of the DFS was also reported to demonstrate good validity and reliability, with a 3-factor structure including specific stimuli, anticipatory fear and avoidance and physiological arousal. The Vietnamese study further supports the cross-cultural applicability of the DFS, while also highlighting that translated versions should continue to be evaluated in different populations and clinical contexts. In the present Turkish validation, the EFA-derived domains were broadly comparable to these international findings; however, the limited CFA fit observed in our study suggests that the structural stability of the Turkish DFS should be interpreted cautiously and further tested in independent samples.^22^ The convergence of these findings across linguistically and culturally different samples supports the cross-cultural stability of the DFS construct and suggests that the Turkish version preserves the conceptual structure of the original instrument. In this regard, the Turkish DFS may contribute to international dental fear research by enabling comparable assessment across Turkish-speaking populations and facilitating the inclusion of Türkiye in cross-cultural studies of dental fear, dental avoidance and patient-centred oral health outcomes.

The internal consistency of the Turkish DFS was excellent. Cronbach’s alpha was 0.95 for the total scale, and the subscale alpha values ranged from 0.81 to 0.95. These values indicate that the Turkish DFS items show strong coherence while still representing distinct dimensions of dental fear. The reliability findings are comparable to those reported in previous validation studies. For example, the Greek validation study reported internal consistency values of 0.96 for the DFS in both study samples,^23^ while the Japanese version reported Cronbach’s alpha values ranging from 0.94 to 0.96.^20^ The Lebanese Arabic version also demonstrated high internal consistency, with a Cronbach’s alpha of 0.93.^21^ These comparisons indicate that the Turkish DFS performs within the expected reliability range for validated DFS versions.

Test–retest reliability was also excellent in the present study. The ICC for the total DFS score was 0.98, and the ICC values for the 3 EFA-derived subscales ranged from 0.96 to 0.98. These findings demonstrate a high degree of temporal stability over a 2-week interval. Previous validation studies have also reported strong test–retest reliability for translated DFS versions. In the Japanese validation, the ICC was 0.92 and Spearman test–retest correlations ranged from 0.89 to 0.92.^20^ The Greek validation reported a DFS test–retest reliability of 0.95,^23^ and the Lebanese Arabic version reported an ICC of 0.92 in a test–retest subgroup.^21^ The present findings therefore provide strong evidence that the Turkish DFS produces stable scores over time.

The Turkish DFS also demonstrated strong convergent validity. The total DFS score showed a strong positive correlation with the Turkish MDAS score (r = 0.83), and all 3 subscales were significantly correlated with MDAS. This finding is expected because both instruments assess dental fear or anxiety, but it should not be interpreted as indicating that they measure identical constructs. The MDAS is a brief screening instrument focused mainly on dental anxiety in selected situations, whereas the DFS provides a broader multidimensional assessment, including avoidance behaviour, physiological arousal and fear of specific clinical stimuli and procedures. This distinction has also been emphasised in the Greek validation study, which noted that the DFS samples a broader range of dental stimuli and physiological responses than the MDAS.^23^ In the same study, the correlation between DFS and MDAS was 0.89, supporting construct validity. Similarly, the Lebanese Arabic validation reported a significant correlation between DFS-A and MDAS-A, supporting convergent validity.^21^ The Romanian validation study also found that the DFS total score and subscales were meaningfully correlated with MDAS.^24^ The strong DFS–MDAS correlation observed in the present study therefore supports convergent validity while maintaining the clinical value of the DFS as a more detailed multidimensional measure.

From a practical perspective, the Turkish DFS should not be viewed as a replacement for the Turkish MDAS, but rather as a complementary instrument when a more detailed clinical profile of dental fear is needed. The MDAS is brief and useful for rapid screening of overall dental anxiety, whereas the DFS provides additional information on avoidance behaviour, physiological arousal, and fear related to specific dental stimuli and procedures. This distinction may be clinically relevant because 2 patients with similar global anxiety scores may differ in their dominant fear pattern; for example, one may primarily avoid appointments, another may show marked physiological arousal, and another may report stimulus-specific fear related to needles, drills, or dental clinic cues. Therefore, the longer format of the DFS may offer added value in clinical and research settings where identifying the components of dental fear is important, while the MDAS remains advantageous for brief screening.

### Clinical implications

The multidimensional structure of the Turkish DFS may have practical value in clinical dental settings. Unlike brief screening tools that primarily provide a global anxiety score, the DFS may help clinicians distinguish between different components of dental fear, including dental avoidance and anticipatory anxiety, physiological arousal during dental treatment and fear of specific dental stimuli and procedures. This distinction may support a more detailed understanding of the dominant fear pattern reported by individual patients. For example, high scores in the anticipatory anxiety and avoidance domain may indicate the need for more careful pretreatment communication and reassurance, whereas high physiological arousal scores may suggest that relaxation-based or behavioural support strategies could be considered. Similarly, high scores on items related to specific stimuli, such as needles or dental drills, may help clinicians anticipate procedure-specific distress and communicate more effectively with patients before treatment.

However, these clinical implications should be interpreted cautiously. The limited CFA model fit, particularly the RMSEA value of 0.10, suggests that the subscale structure should not yet be treated as definitively confirmed for individual-level clinical decision-making. In everyday clinical use, the Turkish DFS may be most appropriately used as a structured aid to identify possible fear domains and guide patient–clinician communication, rather than as a standalone diagnostic or treatment-planning instrument. The present study was designed to evaluate the psychometric properties of the Turkish DFS and did not assess clinical interventions, behavioural management strategies, or treatment outcomes. Therefore, the potential role of DFS subscales in guiding individualised clinical management should be examined in future interventional and outcome-based studies.

### Strengths and limitations

The present study has several strengths. First, the translation and cultural adaptation process followed established methodological guidance and included expert review, pilot testing and content validity assessment. Second, the psychometric evaluation was conducted in a relatively large adult clinical sample and used independent subsamples for EFA and CFA, which strengthens the evaluation of structural validity. Third, the study assessed multiple measurement properties, including internal consistency, test–retest reliability, item-total correlations, split-half reliability and convergent validity with a previously validated Turkish dental anxiety scale. These methodological features provide a comprehensive evaluation of the Turkish DFS.

Several limitations should also be acknowledged. First, the study was conducted in a single university dental faculty setting, which may limit the generalisability of the findings to other clinical settings or community-based populations. In addition, most participants had visited a dentist within the previous 6 months, indicating that the sample may have included individuals who were already engaged with dental care. Therefore, the most fearful individuals, particularly those who avoid or delay dental visits because of severe dental fear, may have been under-represented. Future studies should evaluate the Turkish DFS in more diverse clinical and community-based samples, including individuals with high levels of dental avoidance. Second, although the sample size was adequate for factor analysis, the study included only adult dental patients; therefore, further studies are needed to evaluate the performance of the Turkish DFS in adolescents, older adults, highly fearful dental patients and general population samples. Third, the present study did not establish a diagnostic cut-off score for high dental fear. In contrast, the Lebanese Arabic validation study estimated an optimal cut-off score for identifying dental fear. Therefore, future Turkish studies should examine ROC-based cut-off values and diagnostic accuracy in clinically defined fearful and nonfearful groups. Fourth, although the modified CFA model showed improvement compared with the initial model, the overall model fit remained limited. In particular, the RMSEA value of 0.10, NFI value of 0.86 and CMIN/df value of 3.73 indicate that the 3-factor structure should be interpreted cautiously rather than as showing strong confirmatory support. Therefore, future studies should further examine the structural validity and stability of the Turkish DFS factor structure in larger and independent Turkish samples. Finally, the test–retest interval was limited to 2 weeks; longer-term stability was not assessed.

## Conclusion

The Turkish version of the DFS demonstrated favourable psychometric properties in an adult clinical dental population, particularly in terms of internal consistency, test–retest reliability and convergent validity. The scale showed a theoretically meaningful multidimensional structure; however, the CFA findings indicate that structural validity should be interpreted cautiously and further confirmed in independent samples. These findings support the Turkish DFS as a useful multidimensional instrument for assessing dental fear among Turkish-speaking adult dental patients. Further studies are warranted to evaluate its diagnostic cut-off values and performance in different clinical and community populations.

## Ethics approval

Ethical approval was obtained from the Akdeniz University Clinical Research Ethics Committee (TBAEK-1205).

## Informed consent

Written informed consent was obtained from all participants.

## Author contributions

**Fatih OLUŞ:** Conceptualization, methodology, investigation, data curation, translation and cultural adaptation, writing – original draft, writing – review and editing. **Hüseyin BABUN:** Conceptualization, methodology, supervision, validation, formal analysis interpretation, writing – original draft, writing – review and editing, project administration. All authors have read and approved the final version of the manuscript and agree to be accountable for all aspects of the work.

## Funding

This research did not receive any specific grant from funding agencies in the public, commercial, or not-for-profit sectors.

## Conflict of interest

None reported.

## Appendix Supplementary materials

Image, application 1

## Footnotes

Supplementary material associated with this article can be found in the online version at doi:10.1016/j.identj.2026.109774.
