# Supplementary material for: Psychometric Evaluation of the Turkish Version of the Dental Fear Survey
Source: Int Dent J. 2026 Aug 1;76(5):109774. doi: 10.1016/j.identj.2026.109774 (PMC13453445; doi:10.1016/j.identj.2026.109774)
Supplement: Supplementary file 1 [file mmc1.docx]

**Supplementary File 1.** Final Turkish version of the Dental Fear Survey

**Diş Hekimliği Korku Anketi (DFS)**

| **Bölüm 1** | 1  **Hiç** | 2  **Bir-İki kez** | 3  **Birkaç kez** | 4  **Sık sık** | 5  **Neredeyse her zaman** |
| --- | --- | --- | --- | --- | --- |
| 1.Diş tedavisi korkusu, randevu almayı ertelemenize neden oldu mu? | ☐ | ☐ | ☐ | ☐ | ☐ |
| 2.Diş tedavisi korkusu, randevuyu iptal etmenize ya da randevuya gitmemenize neden oldu mu? | ☐ | ☐ | ☐ | ☐ | ☐ |
| 3.Diş tedavisi sırasında kaslarım gerilir | ☐ | ☐ | ☐ | ☐ | ☐ |
| 4.Diş tedavisi sırasında nefes alışverişim hızlanır | ☐ | ☐ | ☐ | ☐ | ☐ |
| 5.Diş tedavisi sırasında terlerim | ☐ | ☐ | ☐ | ☐ | ☐ |
| 6.Diş tedavisi sırasında bulantı veya mide rahatsızlığı hissederim | ☐ | ☐ | ☐ | ☐ | ☐ |
| 7.Diş tedavisi sırasında kalp atışlarım hızlanır | ☐ | ☐ | ☐ | ☐ | ☐ |

Bu ankette yer alan maddeler, diş tedavisi ile ilişkili çeşitli durumlar, duygular ve tepkilere yöneliktir. Lütfen her bir madde için, hissettiğiniz duygu veya tepkiye en uygun olan seçeneği işaretleyerek (1, 2, 3, 4 veya 5) değerlendiriniz.

| **Bölüm 2**  **Aşağıdaki durumlarda ne kadar kaygı duyuyorsunuz?** | **1**  **Çok rahat** | **2**  **Biraz kaygılı** | **3**  **Orta düzeyde kaygılı** | **4**  **Çok kaygılı** | **5**  **Hasta hissedecek kadar çok kaygılı** |
| --- | --- | --- | --- | --- | --- |
| 8.Diş hekimi randevusu almak | ☐ | ☐ | ☐ | ☐ | ☐ |
| 9.Diş hekiminin muayenehanesine yaklaşmak | ☐ | ☐ | ☐ | ☐ | ☐ |
| 10.Bekleme odasında oturmak | ☐ | ☐ | ☐ | ☐ | ☐ |
| 11.Diş hekimi koltuğuna oturmak | ☐ | ☐ | ☐ | ☐ | ☐ |
| 12.Diş hekiminin muayene odasındaki kokuları duymak | ☐ | ☐ | ☐ | ☐ | ☐ |
| 13.Diş hekiminin odaya girdiğini görmek | ☐ | ☐ | ☐ | ☐ | ☐ |
| 14.Anestezi iğnesini görmek | ☐ | ☐ | ☐ | ☐ | ☐ |
| 15.Anestezi iğnesinin uygulanmasını hissetmek | ☐ | ☐ | ☐ | ☐ | ☐ |
| 16.Matkabı (dental drill) görmek | ☐ | ☐ | ☐ | ☐ | ☐ |
| 17.Matkabın sesini duymak | ☐ | ☐ | ☐ | ☐ | ☐ |
| 18.Matkabın titreşimlerini hissetmek | ☐ | ☐ | ☐ | ☐ | ☐ |
| 19.Diş temizliği (dişlerin kazınması ve parlatılması) sırasında yapılanları hissetmek | ☐ | ☐ | ☐ | ☐ | ☐ |
| 20.Genel olarak düşünüldüğünde, diş tedavisinden ne kadar korkarsınız? | ☐ | ☐ | ☐ | ☐ | ☐ |

Aşağıda, birçok kişinin kaygı veya korku yarattığını ifade ettiği çeşitli durumlar ve şeyler listelenmiştir. Lütfen her biri için sizde ne düzeyde korku, kaygı veya rahatsızlık oluşturduğunu değerlendiriniz. 1 çok rahat hissettiğinizi, 5 ise kendinizi hasta hissedecek kadar yoğun kaygı yaşadığınızı ifade etmektedir.

**Puanlama:** Tüm maddeler 1–5 arasında puanlanır. Toplam DFS puanı 20 maddenin toplanmasıyla hesaplanır ve 20–100 arasında değişir. Yüksek puan daha yüksek diş hekimliği korkusunu gösterir. Türkçe validasyon çalışmasında alt boyut değerlendirmesi şu şekilde yapılmıştır: Maddeler 1, 2, 8, 9 ve 10 “Diş tedavisinden kaçınma ve beklenti anksiyetesi”; maddeler 3–7 “Diş tedavisi sırasında fizyolojik uyarılma”; maddeler 11–20 “Spesifik dental uyaranlar ve işlemlere yönelik korku”.
